# A prospective, single-centre, randomized, double-blind controlled study protocol to study whether long-term oral metronidazole can effectively reduce the incidence of postoperative liver metastasis in patients with colorectal cancer

**DOI:** 10.1186/s13063-023-07628-y

**Published:** 2023-12-04

**Authors:** Rui Qi Gao, Zhen Chang Mo, Hai Kun Zhou, Peng Fei Yu, Wei Dong Wang, Dan Hong Dong, Xi Sheng Yang, Xiao Hua Li, Gang Ji

**Affiliations:** Department of Digestive Surgery, Xi Jing Hospital, Fourth Military Medical University, Xi’an, Shaanxi, China

**Keywords:** Colorectal cancer, Liver metastasis, Metronidazole, Protocol, Therapy

## Abstract

**Introduction:**

Fifteen to 25% of patients with colorectal cancer have combined liver metastases at the time of diagnosis, whereas an additional 15 to 25% will develop liver metastases after curative resection of primary colorectal cancer, with the vast majority (80–90%) of liver metastases unresponsive to curative resection at first. Colorectal cancer liver metastasis is also the leading cause of death in patients with colorectal cancer. In recent years, several studies have demonstrated that intestinal flora, especially *Fusobacterium nucleatum*, plays a crucial role in the development of colorectal cancer liver metastasis, so we hypothesized that long-term metronidazole use could effectively reduce the incidence of postoperative liver metastasis in colorectal cancer patients.

**Methods/design:**

This study is a prospective, single-centre, randomized, double-blind controlled study in which 300 patients will be randomly assigned to the test group or the control group in a 1:1 allocation ratio. The aim of this trial is to demonstrate that long-term oral antibiotics can effectively reduce the incidence of postoperative liver metastasis in patients with colorectal cancer.

**Ethics and dissemination:**

Ethics approval was obtained from the Ethics Committee at the Chinese Ethics Committee of Registering Clinical Trials (ChiECRCT20210229). The results of this study will be disseminated at several research conferences and as published articles in peer-reviewed journals.

**Trial registration:**

Chinese Clinical Trial Registry ChiCTR2100046201. Registered on July 05, 2021.

**Supplementary Information:**

The online version contains supplementary material available at 10.1186/s13063-023-07628-y.

## Introduction

The liver is the most prominent target organ for haematogenous metastasis of colorectal cancer. Fifteen to 25% of patients with colorectal cancer have combined liver metastases at the time of diagnosis, and an additional 15 to 25% will develop liver metastases after curative resection of primary colorectal cancer, with the vast majority (80–90%) of liver metastases unresponsive to curative resection at first. Colorectal cancer liver metastasis is also the leading cause of death in patients with colorectal cancer.

In 2006, a review [1] that summarized relevant articles from 1991 to 2004 concluded that the search for specific bacteria among the normal flora of bacteria is a reasonable way to evaluate the role of bacteria in chronic inflammatory diseases and related cancers. The ability of complex bacterial flora to cause inflammation or cancer may depend on the aggregate activity of multiple components of the flora, rather than a single species, and this ability may be enhanced or diminished with significant changes in species diversity and abundance within the flora. In 2012, an article suggested that chronic inflammation in the gut can affect colorectal carcinogenesis by altering the gut microbial composition to contribute to CRC development [2]. Their study showed that inflammation alters the host physiological status to promote the development of cancer, as seen in colitis-associated colorectal cancer, and identified gut microbiota as a target of inflammation that influences CRC progression. Subsequent studies from multiple cohorts around the world identified a large enrichment of *Fusobacterium nucleatum* in faecal samples of colorectal cancer patients by full-throughput sequencing technology [1, 3–6], and in 2017, Matthew Meyerson et al. [7] validated this by establishing a xenogeneic colorectal cancer mouse model, which confirmed that increased abundance of *Fusobacterium nucleatum* in colorectal cancer patients promoted colorectal carcinogenesis as well as the metastatic process. In 2017 [8], a study found that *Fusobacterium nucleatum* was abundant in colorectal cancer tissues of patients with recurrence after chemotherapy and was correlated with the clinicopathological characteristics of patients. Through bioinformatics and functional studies, *Fusobacterium nucleatum* was found to enhance the chemoresistance of colorectal cancer cells by targeted activation of TLR4 and MyD88 (myeloid differentiation factor) as well as specific microRNAs to activate autophagy pathways, which ultimately alters the chemotherapeutic prognosis in patients. *Fusobacterium nucleatum* has a powerful driving effect on colorectal carcinogenesis, and in addition, as an important intratumoral bacterium, *Fusobacterium nucleatum* is involved in tumour progression and mediates tumour resistance to chemotherapeutic drugs. The mechanism by which *Fusobacterium nucleatum* promotes colorectal carcinogenesis was further investigated by Ge Zhang in 2017 [9], which proposed that exosomes derived from *Fusobacterium nucleatum*-infected cells played a role in promoting cancer initiation and progression. All the above studies have shown that the composition and abundance of gut microbiota play a crucial role in the development of CRC, and the currently known microbiota with distinct effects on CRC tumorigenesis are mainly *Escherichia coli* and *Fusobacterium nucleatum*.

Can we reduce the effect of *Fusobacterium nucleatum* on colorectal carcinogenesis by subjecting patients to long-term metronidazole treatment in the clinical setting? Therefore, we hypothesize that long-term oral metronidazole treatment can reduce the incidence of postoperative recurrence and metastasis. This hypothesis is based on the research progress of the correlation between intestinal flora and the occurrence and development of colon cancer.

A 2017 study by Jing Yuan Fang et al. [8] reported that colorectal tumours with a high Fusobacterium load were more prone to recurrence, and a 2017 study [7] demonstrated that treatment of Fusobacterium-positive human colorectal cancer xenograft mouse models with metronidazole could significantly reduce the Fusobacterium load and inhibit cancer cell proliferation and tumour growth. This conclusion somewhat supports our idea that Fusobacterium-positive tumours may benefit from anti-fusobacterial therapy, such as prolonged metronidazole administration.

Based on an examination of the literature, a review published in 2006, by Carlo et al., proposed that long-term metronidazole supplementation is the therapeutic basis for methylmalonic acid (MMA) and propionic acidemia (PA). In 2014, the Society for the Study of Inborn Errors of Metabolism (SSIEM) designated a panel on recommendations for the diagnosis and treatment of methylmalonic acid (MMA), and propionic acidemia (PA), which suggested metronidazole as one of the recommended therapeutic agents; it should be taken lifelong, on a regimen of 10–20 mg/kg/day for 1–2 or 2–3 weeks per month to avoid the emergence of resistant organisms [10].

The long-term effects of metronidazole use cause serious side effects and have been reported as individual cases in recent years. The probability of developing peripheral neuropathy in patients who continue to take metronidazole for > 4 weeks (taking a total dose of > 42 g) for a long period of time is approximately 17.9% [11, 12]. According to calculations, the blood concentration is approximately 34–45 μg/ml when patients develop peripheral neuropathy, and nearly all patients experience symptom resolution after discontinuing the medication. Therefore, for this study, we set the duration of metronidazole administration to 5 years in our patients, and because anaerobes are extremely sensitive to metronidazole, the dosage was extremely low, and in addition to long-term regulatory follow-up, patients in this protocol only needed to maintain daily blood concentrations greater than 0.01 μG/ml, which can maximize patient safety. At the same time, all the studies with patients who showed drug-related adverse reactions were discontinued, which was included in the ITT set and not included in the PP set.

The aim of this prospective, single-centre, large sample, randomized controlled trial is to demonstrate that long-term oral antibiotics can effectively reduce the incidence of post-operative liver metastasis in patients with colorectal cancer. Secondary objectives included safety and acceptability of the drugs used, treatment-related morbidity and mortality, and relevant follow-up.

## Methods/design

The protocol has been structured according to the Standard Protocol Items: Recommendations for Interventional Trials (SPIRIT 2013 Checklist) (Appendix S1).

### Study setting

**Fig. 1:**
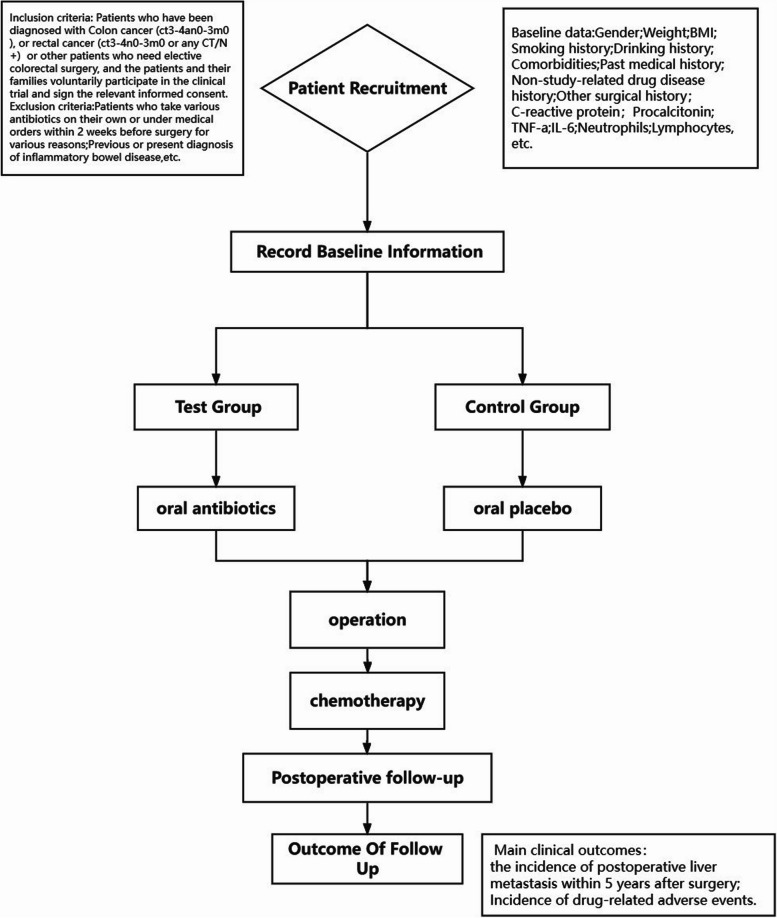
Trial flow chart

This study is a prospective, single-centre, randomized, double-blind controlled study in which 300 patients will be randomly assigned to the test group or the control group in a 1:1 allocation ratio. The study protocol can be accessed at the chictr.org.cn website (ChiCTR2100046201). Figure 1 shows the trial flow chart.

### Eligibility criteria

1. Patients who meet any of the following conditions will be included:
  - Patients of both sexes between 18 and 80 years old;
  - Colon cancer (cT_3-4__a_T_0-3_M_0_ [UICC/AJCC-8th Colon Cancer TNM tumour stage]), rectal cancer (cT_3-4_N_0-3_M_0_ or any CT/N + [UICC/AJCC-8th Rectal Cancer TNM tumour stage]) and patients diagnosed and treated directly with surgery at each study centre;
  - Patients who voluntarily agreed to join this study and signed the relevant informed consent form after verbal discussion about the clinical trial and discussion of the intention of obtaining informed consent.
2. Patients who meet any of the following conditions will be excluded:
  - Taking various classes of antibiotic medications on their own or with medical advice for various reasons within 2 weeks before surgery;
  - Previous diagnosis or current diagnosis of inflammatory bowel disease;
  - Diagnosed with acute intestinal perforation or acute small intestinal diverticulum;
  - Those with ischaemic colitis or infectious colitis were included in the diagnosis;
  - Diagnostic finding of other comorbid local or systemic disease that requires anti-infective treatment;
  - Two and more simultaneous surgeries;
  - Had been diagnosed due to acute and chronic peritonitis or other infectious diseases that require anti-infective treatment in the perioperative period;
  - Any acute physiological disturbance that indicates that the subject requires emergency surgery without elective surgery [e.g., need for preoperative mechanical ventilation, preoperative acute renal failure, preoperative systemic inflammatory release syndrome, and sepsis or septic shock];
  - ASA was rated on a 5-level scale;
  - Immunodeficiency, immunosuppression or autoimmune diseases (e.g., allogeneic bone marrow transplant patients within the last 5 years, taking immunosuppressive drugs, and SLE);
  - The subject refused to sign an informed consent form to participate in the trial;
  - Other scenarios in which normal cooperation with a physician was not possible for personal reasons or the investigator considered it unsuitable to participate in the experiment; and
  - All single-port laparoscopic procedures, transhiatal procedures, and various new surgical modalities need to be used.
3. The terminating study criteria are as follows:
  - Subject's violation of treatment principles after enrolment (such as violation of entry criteria, noncompliance with study treatment medication or surgical arrangements);
  - Subject was inoperable for various reasons after enrolment (reasons to be recorded);
  - Subjects had nonstudy-related complications, such as drug hypersensitivity reactions, after enrolment;
  - Because of the occurrence of adverse events, after the judgement of the investigator, the subject was not considered suitable to continue with the trial (the reason for withdrawal was recorded);
  - Subjects or the subject's legally authorized representative required withdrawal from the study;
  - Subjects developed serious complications or intolerable adverse reactions; and
  - The subject was not available for elective surgery after enrolment due to exacerbation of intercurrent illness or other reasons;

### Interventions

Metronidazole/placebo: Treatment regimens were developed on their own according to each centre’s metronidazole specifications, which required maintenance of metronidazole plasma concentrations for subjects in the experimental group to be > 0.01 μG/ml, and the duration of administration was tentatively set at 5 years (i.e., the time of the outcome of this experiment).

The chemotherapy regimen is eFOLFOX_6_:

Oxaliplatin 85 mg/m^2^ IV infusion for 2 h, D1; leucovorin 400 mg/m^2^ IV for 2 h, D1; fluorouracil 400 mg/m^2^ IV bolus on D1 followed by 1200 mg/m^2^/DX2 continuous IV infusion (total 2400 mg/m^2^ over 46–48 h infusion). This process was repeated every 2 weeks for a total of 24 weeks (Table 1).

**Table 1:** Chemotherapy regimen

Drug name	Dose	Timing of the drugs (a total of 28 days/cycle)							
		Pre-operation	Post-operation						
		D3–D1	D1	D2	D3–D14	D15	D16	D17–D21	D22–D28
Metronidazole/placebo	1 g	√	√	√	√	√	√	√	-
Oxaliplatin	85 mg/m^2^		√	-	-	√	-	-	-
Leucovorin	400 mg/m^2^		√	-	-	√	-	-	-
Fluorouracil	400 mg/m^2^		√	-	-	√	-	-	-
Fluorouracil	2400 mg/m^2^(total)		√		-	√		-	-

### Relevant concomitant care and interventions

There are no restrictions regarding concomitant care during the trial.

### Outcomes

#### Primary outcome

Efficacy will be measured in terms of the incidence of postoperative liver metastasis within 5 years after surgery.

Assessment will be conducted by in-person visits; should the status of the pandemic impact the study with no possibility of performing in-person 5-year follow-up appointments, telemonitoring might be considered using validated tools.

Considering that the dose of metronidazole is predicted in the form of a hypothesis, the incidence of drug-related adverse events will be regarded as one of the main indicators of this study.

#### Secondary outcomes

1. Postoperative complication rates: Postoperative complications will be classified according to their impact and the required management (e.g., medical treatment, reoperation, drainage), as proposed by Clavien–Dindo. Complications will be assessed at the 30-day follow-up; ancillary studies will assess complications at the 60-day and 5-year follow-ups. The Cumulative Complication Index will also be computed to assess the global burden of complications on each patient.
2. Complete postoperative recovery: number of days between admission and when the patient is suitable for discharge.
3. Unplanned secondary surgery
4. Readmission: need for any unplanned readmission after discharge for any reason.
5. 1-year, 3-year and 5-year survival rates
6. Relapse-free survival (RFS)
7. Overall survival (OS)

### Randomization/assignment of interventions

Randomization was stratified by centre. Once informed consent is obtained, randomization will be performed via a centrally managed database. After receiving the patient grouping information, designated researchers will prepare trial drugs or placebo for patients according to the assigned group. In this process, only the researchers are informed about the grouping of patients. They will sign a confidentiality agreement and will not participate in any other links of the trial to ensure that they will not have any impact on the study. After collecting all the data, the designated researcher named the two groups A and B. The analyst will analyse the primary and secondary results without knowing the group name. Only after the analysis of the primary and secondary results can complete blindness be eliminated and ineffective blinding events recorded (for example, the study nurse discloses the patient's specific medication to the doctor).

The nurses provided the patients with oral antibiotics for the experimental group and placebo for the control group in the ward.

### Allocation concealment mechanism

The randomization was performed on a centrally managed database once informed consent was obtained.

### Implementation

The sequence will be generated and forwarded by the computer specialist who created the case report form (CRF) online to implement the online database. By doing so, each investigator can randomize their patients and assign them to the respective group of treatments. The date of randomization will be recorded.

### Blinding (masking)

The blind level was set as double-blind. All patients, doctors, data collectors and analysts will be unaware of the grouping of patients. We do not anticipate any requirement for unblinding but if required, the Trial Manager, Data Coordinator, Implementation Support Facilitators, and Care Home Managers will have access to group allocations and any unblinding will be reported.

### Data collection, management, and analysis

#### Clinical data

Clinical data from patients will be obtained by medical staff and recorded on an online electronic platform (Http://www.medresman.org.cn) and in the CRF table. The sample will be coded, and the patient's identity will be known only by the attending physician. The clinical data will include the following: general patient information, past medical history, past surgical history, laboratory examination results, imaging results, surgery details, postoperative infection rate, incidence of postoperative complications, incidence of anastomotic leakage, and 30-day readmission rate after surgery. The timing and processing of the above-recorded contents will all be reflected in the CRF table, and the laboratory examinations will mainly assess preoperative and postoperative routine blood and inflammatory indicators (Table 2).

**Table 2:** CRF table

Stage	Pre-operation		Intra-operation	Post-operation						
Follow-up period	14–3 days	3–1 days		1–14 days	30 days	1–12 months	12–24 months	24–36 months	36–48 months	48–60 months
Baseline data collected	√	-	-	-	-	-	-	-	-	-
Inclusion and exclusion	√	-	-	-	-	-	-	-	-	-
Sign informed consent	√	-	-	-	-	-	-	-	-	-
Group determination	√	-	-	-	-	-	-	-	-	-
Physical examination	√	-	-	-	√	√	√	√	√	√
Imaging examination	√	-	-	-	-	√	√	√	√	√
laboratory examination	√	-	-	-	√	√	√	√	√	√
First administration	-	√	-	-	-	-	-	-	-	-
Administration	-	-	-	√	√	√	√	√	√	√
Postoperation pathology	-	-	-	√	-	-	-	-	-	-
Safety observation	-	-	√	√	√	√	√	√	√	√
Record adverse events	-	-	√	√	√	√	√	√	√	√
Other works	√	√	√	√	√	√	√	√	√	√

A detailed description of the above data is shown in the CRF table.

#### Sample size calculation basis and formula

A larger sample size is required for this study to provide adequate statistical power.

Based on our current known information and two similar clinical studies on recurrence and metastasis of colorectal cancer (1108 patients enrolled in a berberine hydrochloride clinical trial for colorectal adenoma prevention by Ying Xuan Chen et al., Shanghai Renji Hospital, 2014–2016, Lei Huang et al. from the First Affiliated Hospital of Anhui Medical University during the 2015–2020 clinical trial of aspirin to prevent colorectal cancer metastasis were planned to enrol 3000 patients) and combined with feasibility and study length consideration, we determined the sample size to be 300 patients, experimental group: control group = 1:1, that is, 150 patients in the experimental group and 150 patients in the control group.

#### Statistical analysis

The association with the variables of interest will be compared between groups by means of Pearson’s chi-squared test or Fisher’s exact test, as appropriate and in the case of categorical variables. For continuous variables, Student’s *t* test for independent samples and the Mann–Whitney *U* test will be used for normally and nonnormally distributed variables, respectively.

The analysis for the primary end-point variable (incidence of postoperative liver metastasis) will be performed with an intention-to-treat approach. The absolute difference in terms of incidence will be computed, along with 95% confidence intervals, and will be expressed as relative risk or odds ratio, as appropriate for each outcome.

An interim analysis is planned, once 50% of the patients have completed their treatment (half of the patients per arm). The study can be suspended if the treatment superiority is statistically significant, with a *P* value set at <0.0052. The interim analysis will be performed in agreement with previously reported epidemiological and statistical criteria.

For the secondary variables, a similar approach will be used, using the effect estimate with a 95% confidence interval and the relative risk and odds ratio for categorical variables.

Any potential effects related to centres or surgeons will be assessed by means of mixed models. For the primary end-point variable, a bivariate and multivariate analysis will be performed to identify the potential effects of other variables.

The value for statistical significance at the final analysis was set at *P* < 0.05. Statistical analysis will be performed with SPSS 20. All the deviations from the original statistical plan will be described and justified. All subjects included in the study will be included in the analysis.

### Safety

Adverse events refer to adverse medical events that occur in clinical trial patients after receiving medications. In this study, an adverse event will be considered regardless of whether it is related to the therapy from the time when patients sign the informed consent form to 1 month after the end of treatment. Assessing the nature and determining the severity of adverse events will be conducted in accordance with “expert consensus on diagnostic criteria for postoperative complications of gastrointestinal cancer in China”. To assess the adverse events and their causal relationship to therapy, the investigator will evaluate the possible associations between adverse events and trial medications. The following five criteria will be used to determine the results: the time of occurrence of adverse events coincides with the time of administration, adverse events are related to known adverse reactions of the medication, adverse events cannot be explained by other reasons, adverse events disappear after discontinuing therapy and adverse events are reproduced after medication administration. The results documented as positive, relevant and possibly related were deemed to be adverse reactions. The incidence of adverse reactions will be calculated accordingly. To record, process and report adverse events, the investigator will document any adverse events. Records of adverse events will include a description of adverse events and all related symptoms, time of occurrence, severity, duration, measures taken, the results and final outcomes. The reporting methods and treatment measures for severe adverse events will be classified as severe adverse events if they meet one or more of the following criteria: death, life-threatening (e.g., immediate risk of death), prolonged hospitalization or hospitalization, permanent or severe disability, congenital malformations or defects, some events that have not yet caused death, danger to life or hospitalization but will consider a severe adverse event by a physician if they cause harm to the patient or require medication or surgical treatment to avoid the above situation. For any severe adverse events during the clinical trial, the investigator will file a report of severe adverse events within 24 h and report in writing to the Ethics Committee, the superior authorities and the sponsor. The written report will include the time, severity, duration, measures taken and outcomes of serious adverse events.

### Patient protection/written informed consent forms

Both parties ensure the protection of the patient’s personal records. Except for documents required by law, patient names are not included in any form in tabular reports, publications, or any type of research publication document. Informed consent will be formulated in strict accordance with Chinese laws and regulations. Written informed consent, including all changes made throughout the study, must be preapproved by the Internal Review Board/Independent Ethics Committee before inclusion in the study. Medical staff at each centre will obtain a signature with written informed consent from each patient (if the patient is unable to make their own decision for various reasons, the immediate family will decide on their behalf) prior to any specific activities related to the study. Researchers at each centre will submit and keep original copies of all written informed consent forms signed by patients and provide additional copies to patients or their immediate family members for their records.

### Monitoring

#### Data monitoring

The investigators will not have access to data until the study is completed and data are analysed. A data monitoring committee is established at Xijing Hospital, which will assess the results and safety of the treatments performed at all centres.

Representatives authorized by project undertakers, regulatory departments, and independent ethics committees may visit the centre for inspections, including verifying the original data every year. The purpose of the inspections of the site and personnel is to systematically and independently examine all research-related behaviours and documents, to determine that these behaviours have been managed and that the data have been analysed, recorded and accurately reported in accordance with the research programme, GCP, ICH guidelines and other regulatory requirements.

During the study period, the project undertaker or the supervisor representing the project will regularly contact the research centre for a number of reasons including the following: providing information and technical support; establishing randomized grouping as required; confirming that the investigator complies with the study plan, that data on the CRFs are accurately recorded, and that dosage of drugs being used is checked; and carrying out original data analysis (e.g., the data on CRFs are related to the records of patients in the hospital, and the research will compare these with other records). This requires direct access to the original records of each patient (e.g., clinical charts).

#### Auditing

The project management team will meet once a month to review the progress of the trial. As patients may experience some side effects during long-term administration of metronidazole, the trial steering team at each sub-centre, assigned by the team leader unit, will review the follow-up of subjects at each centre every 2 to 3 months to prevent irreparable harm to patients due to doctors’ or nurses’ errors. The hospital ethics review committees will work together to form an independent data monitoring committee that will review the data from each sub-centre every 3 months and at the time of interim analysis (performed once 50% of the planned sample of patients completed the primary endpoint follow-up )to verify the safety of treatment in both arms.

#### Data ascertainment

For case ascertainment, a screening log will be used, where the following data will be collected:

1. Eligible patients according to the eligibility criteria;
2. Patients who were asked for consent; and
3. Patients who agreed to enter the study.

### Plans for communicating important protocol modifications

Any changes to the trial protocol will first be discussed at an investigator meeting, and based on the discussion the proposed changes will be approved and rejected. If the meeting approves the proposal, a new version of the trial protocol will be prepared by the PI and reviewed and filed with the hospital ethics committee of the PI, who will then inform the centres and send a copy of the revised protocol to the PI to be added to the investigator website archive. Any deviations from the protocol will be reported to the PI and filed by the Data Monitoring Committee and Trial Steering Team.

### Patient protection/written informed consent forms

Both parties ensure the protection of the patient’s personal records. Except for documents required by law, patient names are not included in any form in tabular reports, publications or any type of research publication document. Informed consent will be formulated in strict accordance with Chinese laws and regulations. Written informed consent, including all changes made throughout the study, must be preapproved by the Internal Review Board/Independent Ethics Committee before inclusion in the study. Medical staff will obtain a signature with written informed consent from each patient (if the patient is unable to make their own decision for various reasons, the immediate family will decide on their behalf) prior to any specific activities related to the study. Researchers will keep original copies of all written informed consent forms signed by patients and provide additional copies to patients or their immediate family members for their records.

On the consent form, participants will be asked if they consent to the use of their data if they opt out of the trial. Participants will also be asked if they allow the research team to share relevant data (if relevant) with people from the participating universities or regulatory bodies. This trial will not involve the collection of biological specimens for storage.

### Supplementary Information

**Additional file 1: Appendix S1.** SPIRIT 2013 Checklist.

## Data Availability

Datasets used and/or analysed in this study are available from the corresponding authors on reasonable request.

## Abbreviations

CRC: Colorectal cancer
MMA: Methylmalonic acid
PA: Propionic acidemia
SSIEM: The Society for the Study of Inborn Errors of Metabolism
ITT: Intention-to-treat
PP: Per-protocol
